# Drowning

**Published:** 1879-01

**Authors:** 


Drowning.—There is only one safe way of attempting to save a person who is drowning, and that is to approach him from behind and clasp him around the body in such a way that your arms will be at the elbow bend, then he cannot grasp you; if this is not practicable, from the fact that you must use one arm to sustain yourself in the water, catch hold of him by the hair of the head and hold him at arm's length. If you are thrown in the water and are tired from swimming, throw yourself on your back, with the toes and nose out of the water and the hands toward the bottom clasped; one may rest for an hour in this way. These things should be practised in learning to swim.

